# Applying Complexity Theory to a Hospital Complex Patient Care Program

**DOI:** 10.5334/ijic.5634

**Published:** 2021-05-18

**Authors:** Felice Borghmans, Venesser Fernandes, Harvey Newnham

**Affiliations:** 1 Monash University, AU

**Keywords:** Complexity, systems, information, integration

## Abstract

Increasingly, complexity science concepts are informing health care design and practice. The present paper describes the implementation of early complexity science principles in a Complex Care Program with the aim of strengthening the provision of integrated care. Grounded in cybernetic network theory, Stafford Beers Viable Systems Model [1] provided the guiding principles for the programs redesign. The Viable Systems Model with its broadly applicable principles [1], is now the conceptual model of information management in the program. Beers framework has enabled a relatively small number of clinicians to coordinate care for a large cohort of patients with significant clinical complexity, and a multitude of providers, in the community setting.

## Introduction

Our Hospitals Complex Care Program (CCP) provides care coordination and clinical support for people with chronic health and psychosocial complexities. In 2016 we undertook a service redesign to augment the programs capacity for responsive, well-integrated, patient care. The changes made were informed largely by Beers Viable Systems Model (VSM), an approach grounded in early complexity theory principles of cybernetic network theory [2]. This paper describes the programs change journey of translating generic complex systems principles into a healthcare practice context.

## Overview of the Care Context

Public hospital run CCPs were introduced in the early 2000s to ease hospital demand pressures [3], by reducing so-called avoidable hospital presentations [4,5]. Their objective is to improve the coordination and integration of services for people with chronic health conditions and biopsychosocial complexities [3,6]. Our CCP employs approximately 55 healthcare professionals of which just over half are nurses, about one quarter are social workers and case managers, and the rest are allied health professionals and medical doctors. Currently, the CCP coordinates care for 300 or more patients at a time, largely in the community setting.

## Initial observations: The paradox of care integration and model fragmentation

**Figure 1:**
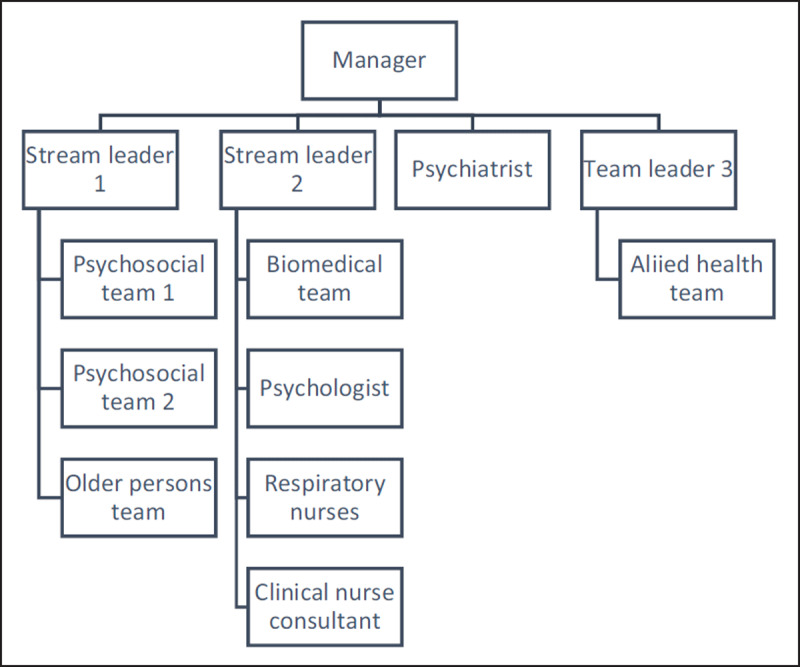
The CCP structure prior to redesign.

Prior to its redesign, the CCP structure consisted of numerous small services, each with an area of expertise. Some teams specialised in psychosocial issues, others were biomedically orientated, one team focused on the care of older persons, and several independent clinical experts held small caseloads. This workforce design resulted from the project-like nature of CCPs in the early 2000s, that saw a gradual implementation of various approaches [6]. See ***Figure 1***.

The incremental nature of the programs development had produced a fragmented service structure that stood in contrast to the CCPs core principles of care integration [7]. The program structure fostered siloed work practices that acted as a barrier to well-coordinated healthcare. Additionally, while systems of information sharing and collaboration are fundamental to integrated care practices [8], the CCPs information systems were disjointed. The program operated two client databases, and most patient information was stored separately to the hospitals main electronic medical record (EMR). Hence, pertinent CCP patient information was inaccessible to hospital clinicians. In addition, each small team had unique documentation templates, which fragmented information even within the CCP.

A consistent purpose and role scope are critical to the identity and strategic intent of a service and should differentiate it from other services [1]. In contrast, a multitude of teams, each with distinctive orientations meant the CCP lacked a cohesive identity. Furthermore, because patient eligibility for the program was determined at the clinician level, access to the CCP was highly variable, which confused referrers to the program.

Regarding care delivery, the CCP employed a key worker model, which is thought to foster client-carer engagement [9]. However, the nebulous issues encountered in complex care demand collaborative problem solving and the sharing of expertise [10]. Hence, the CCPs key worker model required augmentation to foster dynamic capabilities [11] across the workforce, and to strengthen oversight for patients with significant medical or psychosocial complexity.

In summary, the design of the CCP constrained its capacity to manage effectively both the internal and external informational complexity to which it was exposed. As a result, it was unclear if the CCP was effective in achieving its objectives of safe, effective, and well-integrated care and the reduction of potentially preventable hospital attendances.

## Matching the model design to its purpose

In researching strategies by which to manage systemic complexity we came across Beers VSM [1]. The VSM purports to promote effective and adaptive system responsiveness to internal and external informational complexity, with general applicability across systems [1]. The VSM was adopted as the starting point for a redesign of the CCP and it has since become an embedded component of the programs operational and clinical practice framework.

## The VSM models complexity principles and their operational application in the CCP

**Figure 2:**
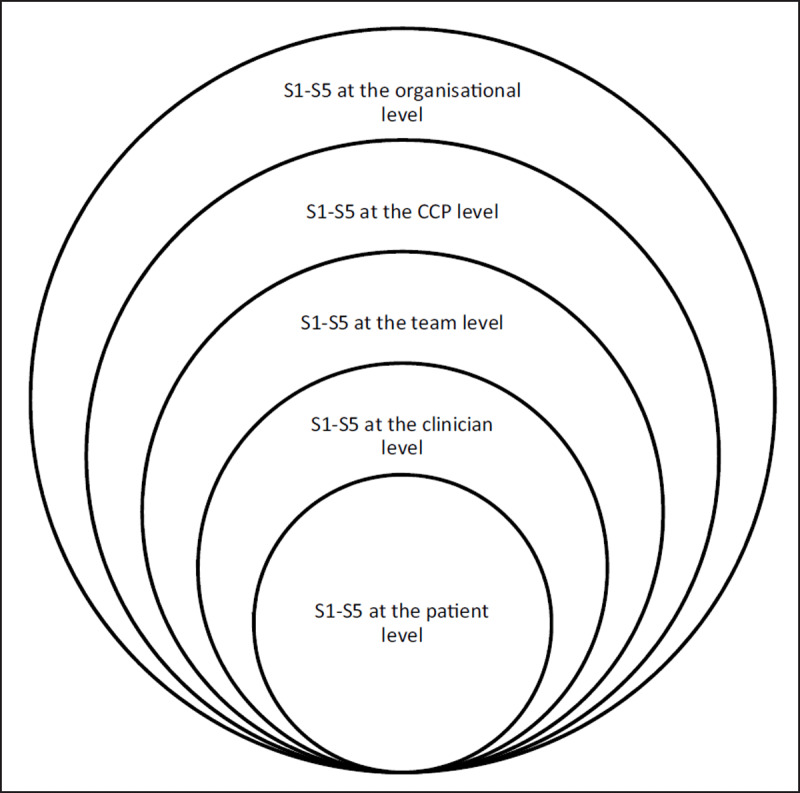
The nested systems design adapted from Beers VSM.

Healthcare programs deal with a vast quantity and variety of information, thus managing this complexity is a central concern [12]. Furthermore, information accessibility is crucial to well-integrated care coordination [8]. The VSM supports attenuation, augmentation, and organisation, to optimise information coordination and accessibility [1,13]. The VSM framework consists of five co-dependent functions represented by the symbols S1 through to S5, where each function (S) is discrete [14], and each sub-component of a system consists of all five functions. As such, subsystems are nested within larger systems as an integrated arrangement of information sharing and management (see ***Figure 2***). In the CCP, individual staff and teams represent subsystems, nested within the larger system; the CCP itself.

S1 represents the function of production that is aligned to the systems core purpose [2]. The CCPs purpose is complex healthcare coordination and integration [3]. Through staff consultation the programs purpose, and the role scope of clinicians, was articulated and distilled into a unified service identity, tied to its eligibility criteria. The programs identity was further strengthened by joining with the hospitals central point of access for ambulatory services. This had the effect of eliminating variable decision-making about patient eligibility to the service and reducing referrer confusion.

S2 is the coordination function of a system [2]. The CCP fully integrated with the hospitals EMR to foster improved oversight and coordination of care across the hospital system. Furthermore, the CCPs multitude of documentation types were consolidated into generic templates for use across the program. The standardised formats have made it easier for clinicians to find pertinent information, and improved consistency in documentation. In addition, while the key worker approach has been largely retained, the CCP has strengthened its model of interdisciplinary care through the adoption of Snowdens Cynefin Framework of decision-making [10]. This flexible team-oriented approach to deliberation is ideally suited to the nebulous issues experienced by patients in the CCP [10].

**Figure 3:**
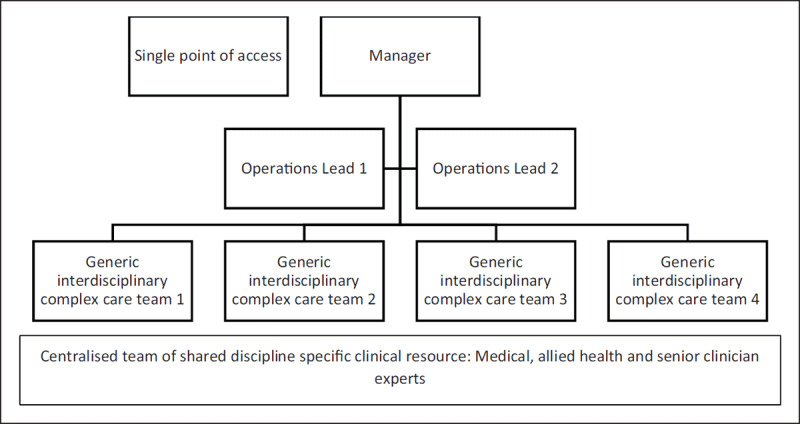
The redesigned workforce structure of the CCP.

Beers S3 function represents reflexive internal regulation in response to changes within or external to a system [2]. The siloed workforce configuration of the CCP constrained S3 capability by partitioning decision making and the sharing of resources. It required a workforce restructure to augment S3 functionality within the service (see ***Figure 3***). The workforce has been reconfigured into four generic interdisciplinary complex care teams. The teams are supported by senior clinicians whose role it is to match clinical resources to patient needs. The generic teams are assisted by area operations leads who in turn are supported by the CCP manager. A fifth centralised team, made up of the CCPs rarer clinical resources, provides individualised discipline specific expertise across the complex care teams.

Reflexive service delivery regulation has been further augmented by redesigning roles across the entirety of the service. Each role has a defined practice scope yet is maximally autonomous within that scope, to foster adaptive decision making. Furthermore, in Beers VSM, the bounded identity and autonomy of functions is coupled with a robust network of communication, to support flexible, responsive care [1]. Hence, a detailed communication framework now underpins the CCP model. A set of targeted meeting types (such as morning huddles, weekly care planning) promotes timely information exchange so that the status of patients, and the configuration of their care team, is known to relevant parties and readjusted as needed.

The S4 function of the VSM accounts for intelligence; knowledge of changes occurring inside and outside of a system that may require internal adaptation to stay relevant [2]. S4 underscores the need for robust communication within the program to remain contemporary and responsive to community and organisational needs. Since healthcare occurs in a dynamical environment [15,16] the timely acquisition of critical information at the right level of decision-making enables proactive adjustments to care [1]. Therefore, maximising clinician autonomy, and fostering robust feedback, are vital elements of adaptive healthcare in the CCP [13].

Finally, S5 represents the systems executive function; its direction setting and future planning, which requires all the other elements, S1 to S4, to align and to communicate [14]. For the CCP, S5 requires the cohesive orientation of all team members to the programs funded objectives: The reduction of potentially preventable hospital attendance through effective coordination of care for complex biopsychosocial health needs [3]. Yet, to be meaningfully adopted the objectives must align with clinicians aims and values also [17]. Thus, the knowledge contribution of clinicians skilled in working with patients that have complex health needs has been critical to the implementation and continuation of changes to the program.

## Implications for Practice

The VSM incorporates invariant elements that, in combination, enabled us to better integrate interdisciplinary care effectively [1]. We recommend its application in programs seeking to manage the complexity of caring for patients with diverse needs and dealing with multiple stakeholders across different parts of the healthcare sector.

## Conclusion and Recommendations

Complexity thinking, informed by Beers VSM [1] has improved the integration of information and care across the CCP. However, one cannot set and forget work of this nature. Complexity thinking exposes the dynamical nature of myriad systems informing healthcare [12]. Healthcare models demand constant attention and reflexive engagement to stay relevant [1]. Never-the-less, our experience to date suggests complexity thinking may be applicable in similar settings of care.
